# Self-Propagating Combustion Triggered Synthesis of 3D Lamellar Graphene/BaFe_12_O_19_ Composite and Its Electromagnetic Wave Absorption Properties

**DOI:** 10.3390/nano7030055

**Published:** 2017-03-03

**Authors:** Tingkai Zhao, Xianglin Ji, Wenbo Jin, Wenbo Yang, Xiarong Peng, Shichang Duan, Alei Dang, Hao Li, Tiehu Li

**Affiliations:** State Key Laboratory of Solidification Processing, Shaanxi Engineering Laboratory for Graphene New Carbon Materials and Application, School of Materials Science and Engineering, Northwestern Polytechnical University, Xi’an 710072, China; wenbo_jin@126.com (W.J.); yangwenbo@163.com (W.Y.); pengxiarong@163.com (X.P.); duanshichang@163.com (S.D.); dangalei@nwpu.edu.cn (A.D.); lihao@nwpu.edu.cn (H.L.), litiehu@nwpu.edu.cn (T.L.)

**Keywords:** 3D lamellar graphene, BaFe_12_O_19_, self-propagating combustion, electromagnetic wave absorbing property

## Abstract

The synthesis of 3D lamellar graphene/BaFe_12_O_19_ composites was performed by oxidizing graphite and sequentially self-propagating combustion triggered process. The 3D lamellar graphene structures were formed due to the synergistic effect of the tremendous heat induced gasification as well as huge volume expansion. The 3D lamellar graphene/BaFe_12_O_19_ composites bearing 30 wt % graphene present the reflection loss peak at −27.23 dB as well as the frequency bandwidth at 2.28 GHz (< −10 dB). The 3D lamellar graphene structures could consume the incident waves through multiple reflection and scattering within the layered structures, prolonging the propagation path of electromagnetic waves in the absorbers.

## 1. Introduction

Electromagnetic wave absorbing materials have attracted much attention in commercial and military applications and it is significant to produce microwave absorbing materials with excellent absorption properties [1,2]. High performance microwave absorbing materials can be widely used in many fields such as medicine [3,4,5], electronic safety and national defense security due to their broadband high-performance absorbing ability, low density and thin thickness. 

The free-standing three-dimensional (3D) graphene synthesized by self-assembled method with the characteristics of broad frequency bandwidth, ultrahigh compressibility and adjustable electrical conductivity [6], could be used as a high performance microwave absorber. The broader the bandwidth is, the better performance the material presents. This unique structure greatly shortens the impedance gap, weakening the reflection and scattering, which help the incident wave pass through the entire foam, and improve the capacity of radiation absorption. However, the weakness of low stability and large load content of single material seriously hinder the practical application. The new microwave absorbing composites obtained by the compound with other complementary materials can be used to a great extent for improving its comprehensive performance [6]. As a dielectric loss material, graphene usually compound with magnetic loss materials in order to obtain good electromagnetic wave absorbing properties. BaFe_12_O_19_ is widely used as magnetic substrate because of its high coercivity, excellent magnetization, good magnetocrystalline anisotropy, easy preparation with low cost, and adjustable properties [7,8,9,10]. Especially, the extraordinarily high Curie temperature [11] ensures the thermostability of BaFe_12_O_19_. Nevertheless, how to obtain ultra-light and economic graphene/BaFe_12_O_19_ composite has become the essential issue in the preparation process of the composite material. 

Here, we report a potential use of the self-propagating combustion [12] process in preparing microwave absorption materials. The composite synthesis was carried out by self-propagating combustion, utilizing the self-heating and self-conduction effect of high reactant heat among substrates [13]. Herein, graphite was first pre oxidized in order to activate the graphite layers and then 3D lamellar graphene/BaFe_12_O_19_ composite was prepared by self-propagating combustion, triggered by the exothermic oxidation-reduction reaction between citric acid and nitrate. The high temperature generated by self-propagating combustion could vaporize the oxygen-containing functional group of graphite and promote the formation of BaFe_12_O_19_ crystal. Therefore, graphite sheets that can take advantage of the energy are largely expanded into large amounts of lamellar graphenes due to the sudden gasification and stupendous volume expansion effect. The two-step process of preparing stabilized 3D lamellar graphene and embedding BaFe_12_O_19_ between the graphite layers to synthesize the composite material is completed at one time, which lead to simple operation procedure. In this paper, the self-propagating combustion triggered synthesis process of 3D lamellar graphene/BaFe_12_O_19_ composites has been studied, which has not been reported before.

The 3D independent graphene in this composite has a wide frequency band and high dielectric constant characteristic. Meanwhile, BaFe_12_O_19_ with high permeability and coercivity can easily widen absorption bandwidth and further expand reflection loss as well as remedying the tremendous gap between low permeability and high complex permittivity. Such superiorities of self-propagating combustion with short reaction time; little pollution, high temperature and high energy triggered by low temperature and low energy; the capability of chemical exothermic maintenance by itself; and high quality of purity of the product attract much attention [14,15].

## 2. Experimental

**Figure 1:**
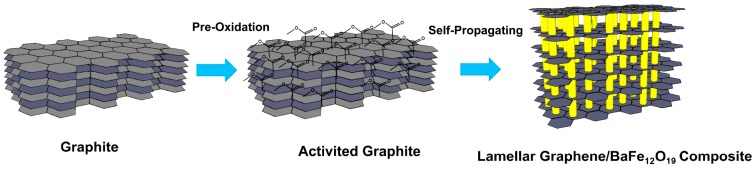
Schematic of the fabrication process of 3D lamellar graphene/BaFe_12_O_19_ composite.

**Figure 2:**
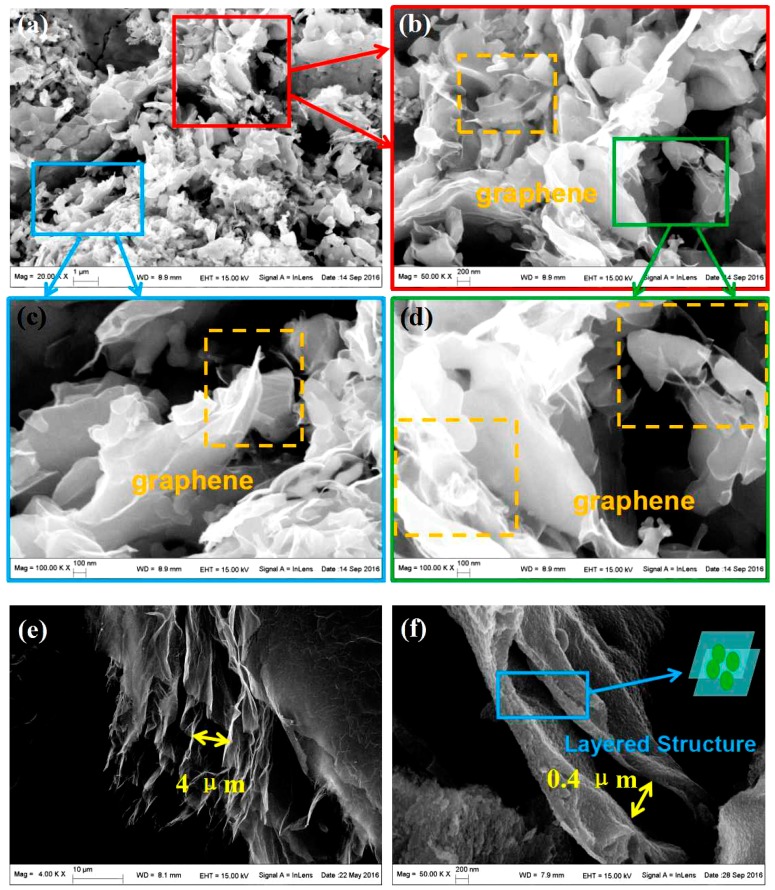

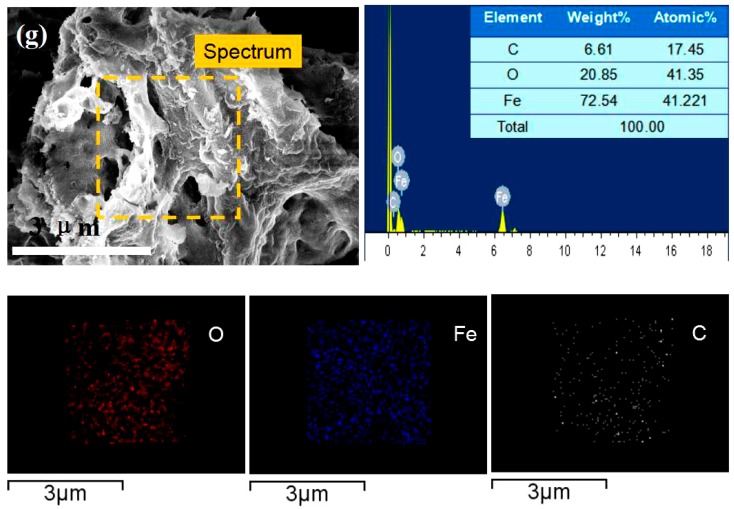
SEM images of 3D lamellar graphene/BaFe_12_O_19_ composite: (**a**) SEM image of 3D lamellar graphene/BaFe_12_O_19_ composite; and (**b**–**d**) enlarged parts of (**a**). The transparent sheets in the yellow rectangular blocks are graphene. (**e**) The activated graphite sheets treated at 1000 °C for eight seconds as comparison; and (**f**) activated graphite sheets treated by self-propagating combustion. The layers of the graphite are largely expanded and filled with BaFe_12_O_19_. The distance between the layers varies from 0.4 to 1 μm. (**g**) The EDS analysis and elemental mapping of the lamellar graphene/BaFe_12_O_19_ composite.

The schematic diagram of fabrication process of 3D lamellar graphene/BaFe_12_O_19_ composite is presented in Figure 1. The synthesis process contains two steps: first, graphite was pre oxidized by concentrated sulfuric acid/concentrated nitric acid/KMnO_4_ in order to activate the graphite layers. Second, 3D lamellar graphene/BaFe_12_O_19_ composite was prepared by self-propagating combustion using activated graphite sheets and citric acid compound with Fe(NO_3_)_3_ and Ba(NO_3_)_3_ as precursor at 350 °C. The formation of 3D lamellar graphene structures from graphite mainly based on two effects occurred in the self-propagating combustion reaction process. The self-propagating combustion that was triggered at lower temperature can produce tremendous heat to continue the reaction and form the crystal structure. The temperature during the reaction increased to about 1500 °C. Thus, the high temperature and tremendous heat can gasify the functional groups on the graphite layers. The instantaneous gas flow could easily expand the graphite layers into multilayered graphene. Furthermore, for the second aspect, the self-propagating combustion reaction also has volume expansion effect. The precursor firstly infiltrate into the graphite layers (Figure 2f–h) as liquid state, which would have huge volume expansion effect during the reaction. The graphite layers could be largely expanded due to the volume expansion of BaFe_12_O_19_ during the reaction process. Thus, the 3D lamellar graphene structures could be formed owing to the synergistic effect of the tremendous heat induced gasification as well as the huge volume expansion effect. The schematic of the fabrication procedure is presented in Figure 1.

### 2.1 Activation of Graphite Sheets

The as-provided natural flake graphite (NFG) was activated by concentrated acid for the later reaction. First, 1 g NFG was mixed with 4 g concentrated sulfhuric acid, 2 g concentrated nitric acid and 0.8 g KMnO_4_. Then, the resulting mixture was continuously stirred for 30 min to make the graphite sheets more thoroughly activation with the concentrated acid. Last, the sufficiently activated graphite sheets were washed by distilled water to pH 6–7, and the activated graphite sheets were dried for 24 h. 

### 2.2 In Situ Synthesis of Lamellar Graphene/BaFe_12_O_19_ Composite

The 3D lamellar graphene/BaFe_12_O_19_ composite was in situ prepared by self-propagating combustion method using the activated graphite sheets and citric acid mixed with Fe(NO_3_)_3_ and Ba(NO_3_)_3_ as precursor. The mass ratio of Fe(NO_3_)_3_, Ba(NO_3_)_3_ and citric acid is 10:1:(6–15). The mass fraction of activated graphite sheets are various from 10 to 40 wt %. The ingredients were first dissolved into distilled water and then ammonium hydroxide was added to adjust pH = 7. The obtained colloidal sol was continuously heated and stirred at 350 °C to evaporate water and give rise to the self-propagating combustion process. After the combustion reaction, 3D lamellar graphene/BaFe_12_O_19_ composite was prepared. The sample powders were collected and cleaned for further test and application.

### 2.3. Characterizations

High resolution transmission electron microscope (HR-TEM, Tecnai G^2^ F30, FEI: Hillsboro, OR, USA), field emission scanning electron microscope (FESEM, JSM-6700F, JEOL, Peabody, MA, USA), X-ray diffraction spectrometer (XRD, X’Pert PRO MPD, PANalystal, Almelo, The Netherlands, λ = 0.154 nm) and the Raman spectrometer (514 nm, LabRAM HR800, HORIBA JOBIN YVON, Kyoto, Japan) are used for qualitative analyses on the morphology, structural characteristics and component composition of all samples. The as-obtained samples that were used for the characterization of electromagnetic wave absorbing properties were prepared by mixing 10 wt % 3D lamellar graphene/BaFe_12_O_19_ composite with 90 wt % molten paraffin, then made into a doughnut-shaped sample (*Φ*_out_: 7.03 mm, *Φ*_in_: 3.00 mm). The complex permittivity (*ε′, ε″*) and permeability (*μ′, μ″*) components as the function of frequency of a sample are measured using a MS4644A vector network analyzer (Anritsu, Kanagaw, Japan, 10 MHz–40 GHz).

## 3. Results and Discussion

Figure 2a–d shows SEM images of 3D lamellar graphene/BaFe_12_O_19_ composite, where in Figure 2b–d shows enlarged parts of Figure 2a. The transparent sheets in the yellow rectangular blocks are composed of graphene. The particle size containing graphene/BaFe_12_O_19_ is about 1–4 μm in diameter. The transparent sheets are made up of graphene sheets, which spread and exist everywhere in Figure 2. The graphene sheets and BaFe_12_O_19_ mixed with each other. It can be assumed that the graphite layer was expanded by the synergistic effect of the heat releasing and volume expansion. Thus there are quantities of transparent graphene sheets on the edge or surface of BaFe_12_O_19_ particles. Figure 2f further proves the volume expansion effect. BaFe_12_O_19_ is embedded inside the graphite layers. The expanded graphite layers are coated and surrounded with BaFe_12_O_19_ particles. The liquid BaFe_12_O_19_ precursor penetrates into the activated graphite layers before the reaction start and the huge volume expansion effect drastically separate the graphite layers. The layer distance varies from 0.4 to 1 μm. The difference of the layers may be due to the inhomogeneous volume expansion reaction. As a comparison, Figure 2e shows expanded graphite (EG) which formed from activated graphite sheets after treatment at 1000 °C. The expansion of the layers mainly is due to the instantaneous gasification of the functional group joint inside the layers, which inflates the graphite to separate the layers. The distance between the layers is about 4 μm, which is larger than that of the graphite/BaFe_12_O_19_. There are several reasons for this issue. First, although the temperature of the reaction zone for graphite/BaFe_12_O_19_ is near 1000 °C, as in Figure 2e, the expansion can be inhibited due to the existence of BaFe_12_O_19_. In Figure 2e, there is no solid resistance such as BaFe_12_O_19_. Besides, although the self-propagating combustion releases tremendous heat, the limited reaction time would partially influence the expansion process. Figure 2g is the EDS analysis and elemental mapping of 3D lamellar graphene/BaFe_12_O_19_ composite. The content of Ba element is too small so that it is hard to be detected. The weight ratios or the atomic ratios of the C, O, and Fe are labeled in the figure. From the elemental mapping, the distributions of C, O, and Fe are homogeneous, indicating the graphene sheets and BaFe_12_O_19_ are mixed uniformly during the reaction process. 

**Figure 3:**
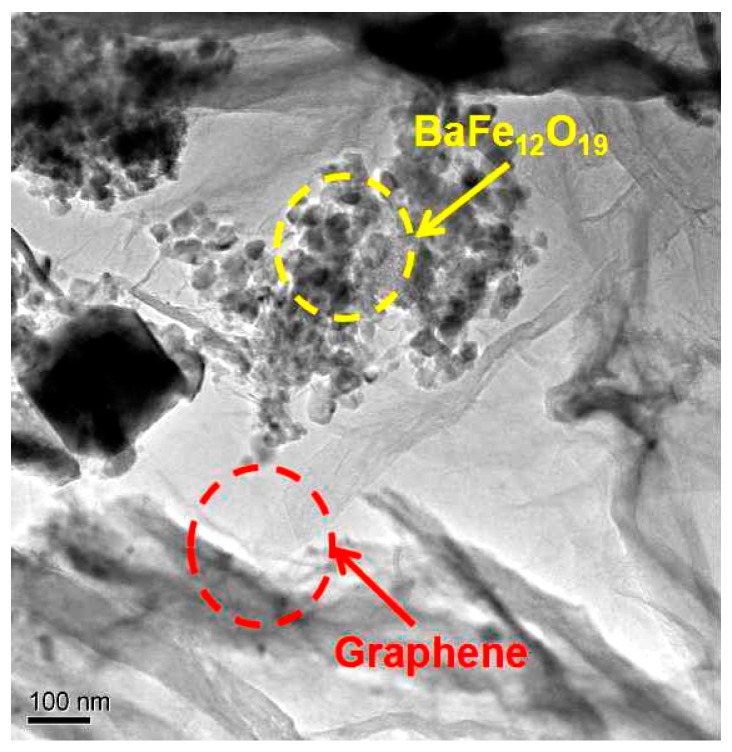
TEM image of 3D lamellar graphene/BaFe_12_O_19_ composite.

Figure 3 is a TEM image of 3D lamellar graphene/BaFe_12_O_19_ composite. Figure 3 shows the micromorphology of the mixture of graphene and BaFe_12_O_19_. The graphene sheets were folded together with wrinkle and embedded with BaFe_12_O_19_ particles. BaFe_12_O_19_ particles mainly have two kinds of shapes. One part of the BaFe_12_O_19_ particles with average size of 50 nm (circled with yellow line), and the others are rectangular-like shape. The graphene sheets interlinked BaFe_12_O_19_ particles for increasing the dielectric loss in the composite. The 3D structure of lamellar graphene/BaFe_12_O_19_ composite is beneficial to the pass through of electromagnetic wave in different directions. 

**Figure 4:**
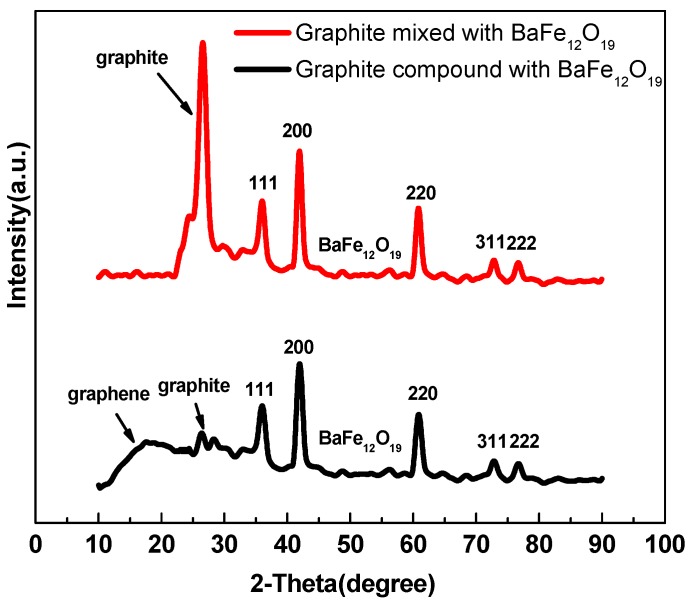
XRD spectra of graphite mixed with BaFe_12_O_19_ and 3D lamellar graphene/BaFe_12_O_19_ composite.

Figure 4 shows XRD patterns of graphite mixed with BaFe_12_O_19_ and 3D lamellar graphene/BaFe_12_O_19_ composite. The BaFe_12_O_19_ peaks labeled with crystal indices in the two curves are coincident. The spectrum of 3D lamellar graphene/BaFe_12_O_19_ composite shows the graphene peak at around 18°, proving the formation of graphene and the graphite peak at around 26°. Compared with the graphite peak in the upper curve, the peak intensity of graphite is far lower in that of lamellar graphene/BaFe_12_O_19_ composite, indicating that there is only a little residual graphite after the self-propagating combustion process. The BaFe_12_O_19_ spectrum has sharp peaks showing the good crystallinity. 

**Figure 5:**
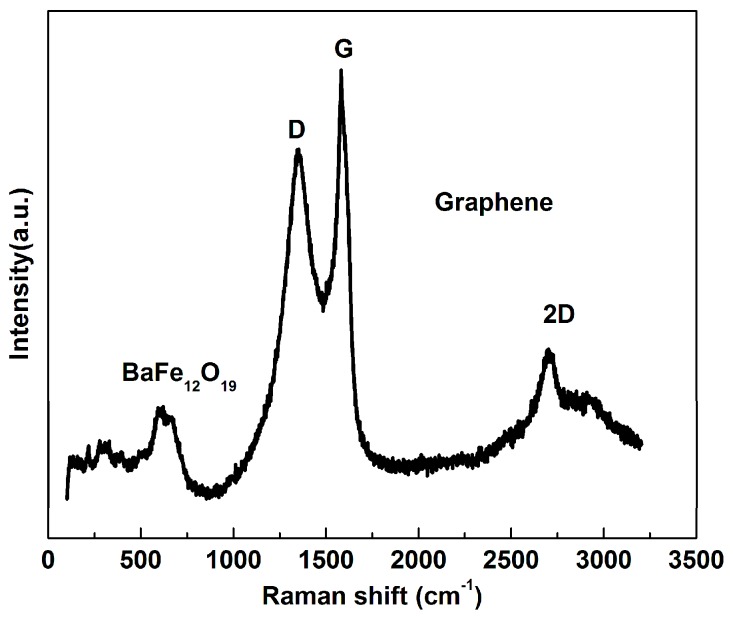
Raman spectrum of 3D lamellar graphene/BaFe_12_O_19_ composite.

Figure 5 is Raman spectrum of 3D lamellar graphene/BaFe_12_O_19_ composite. The spectrum has two obvious peaks centered at 1324.7 and 1593.2 cm^−1^, namely D and G modes of graphene. The D peak at 1324.7 cm^−1^ stems from a symmetry-lowering effect owing to the defects of graphene sheets. The G peak at 1593.2 cm^−1^ indicates the graphite crystallinity of carbon material. The ratio of *I*_D_ to *I*_G_ is used to characterize the structure integrity of carbon materials. 3D lamellar graphene/BaFe_12_O_19_ composite shows both the characteristic peak of BaFe_12_O_19_ (labeled in the image) and graphene. The presence of 2D peak indicates the existence of graphene. Therefore, from above characterization and analysis, the graphene/BaFe_12_O_19_ composite have a 3D lamellar structure and the BaFe_12_O_19_ particles were embedded inside the graphene sheets after the self-propagating combustion process.

**Figure 6:**
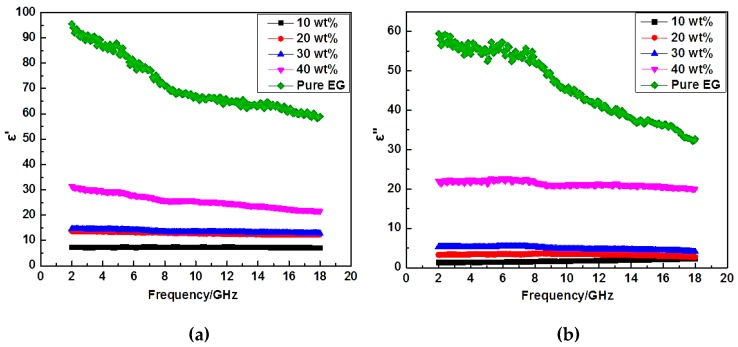

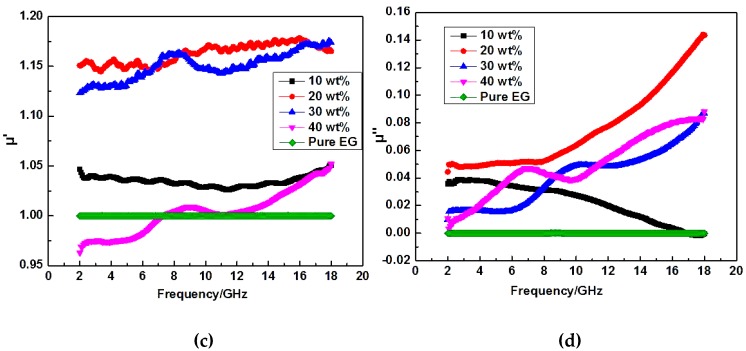
Complex permittivity (*ε′, ε″*) and permeability (*μ′, μ″*) spectra of 3D lamellar graphene/BaFe_12_O_19_ composites (with different graphene mass fraction) vs. frequency: (**a**,**b**) complex permittivity; and (**c**,**d**) the complex permeability.

**Figure 7:**
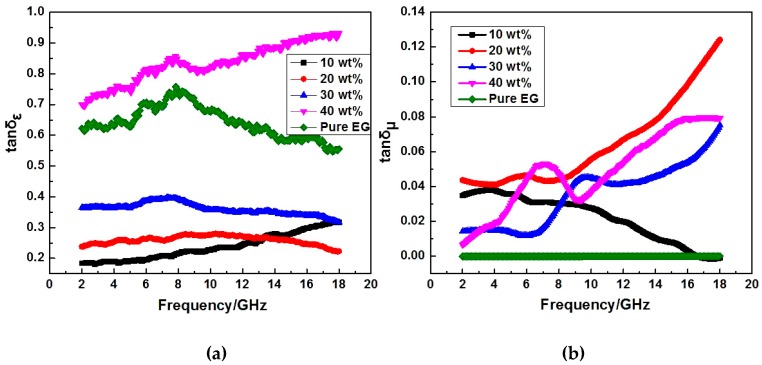
Dielectric loss tangent (tanδ_ε_) and magnetic loss tangent (tanδ_μ_) of 3D lamellar graphene/BaFe_12_O_19_ composites (with different graphene mass fraction) vs. frequency: (**a**) the dielectric loss tangent (tanδ_ε_); and (**b**) the magnetic loss tangent (tanδ_μ_).

**Figure 8:**
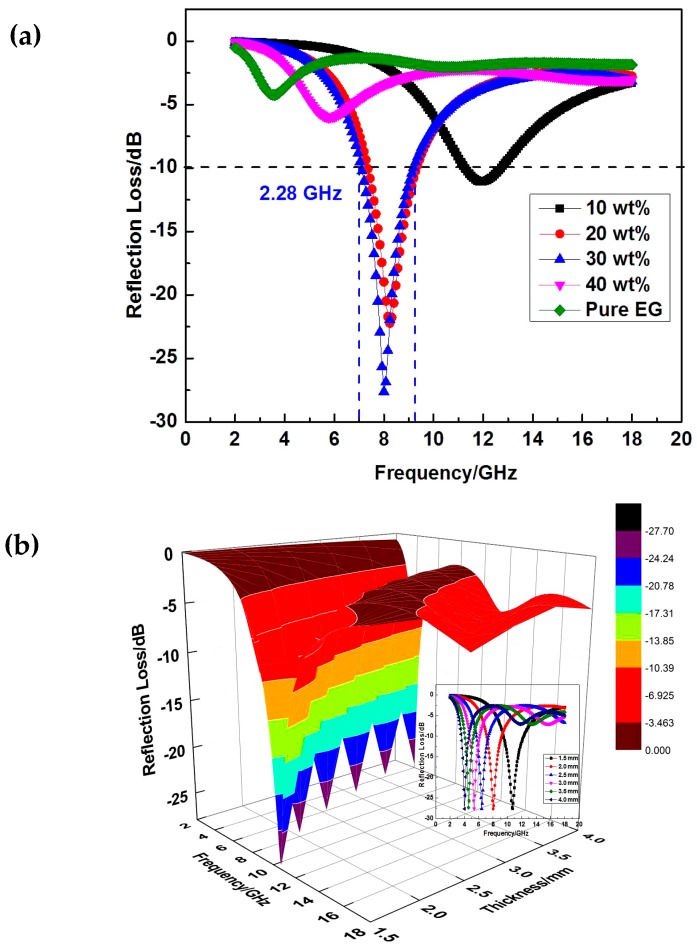
Electromagnetic wave absorbing properties of lamellar graphene/BaFe_12_O_19_ composites with different: (**a**) mass fraction of graphene (2 mm); and (**b**) thickness of the samples (30 wt %).

**Figure 9:**
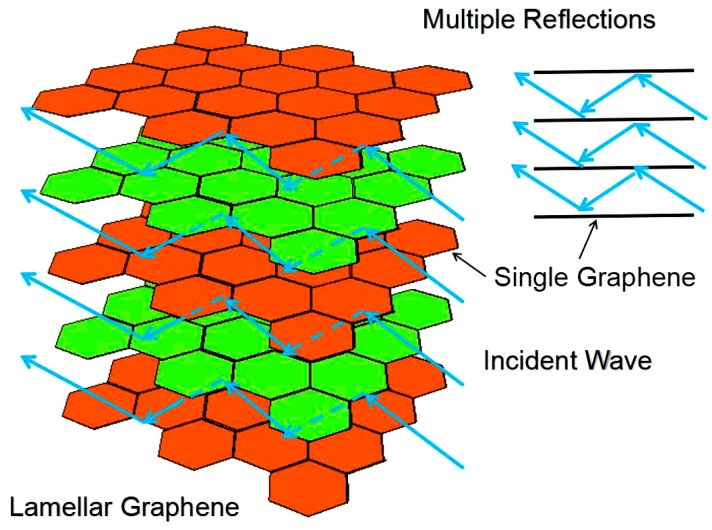
Electromagnetic wave absorbing mechanism schematics of 3D lamellar graphene/BaFe_12_O_19_ composites.

The complex permittivity (*ε′, ε″*) and permeability (*μ′, μ″*) curves of 3D lamellar graphene/BaFe_12_O_19_ composites (with different graphene mass fraction) vs. frequency are shown in Figure 6a–d. The permittivity and permeability are used for characterization of dielectric and magnetic loss properties of the electromagnetic wave absorbing materials. The real permittivity (*ε′*) and permeability (*μ*′) represent the storage ability of electromagnetic energy, whereas the imaginary permittivity (*ε*″) and imaginary permeability (*μ″*) are connected with the energy dissipation and magnetic loss, respectively [16]. In Figure 6a,b, the dielectric constant (*ε′*) decreases slightly with frequency increasing, which is also observed by other researchers [17]. According to Koops theory based on the Maxwelle Wagner model for the inhomogeneous double layer dielectric structures [18], the well conducting grains are separated by poorly conducting grain boundaries. Here, the interaction within BaFe_12_O_19_, graphene and paraffin wax plays an important role in the formation of the grain boundaries. At low frequencies, the grain boundaries are more effective than the conducting grains. Owing to the high resistance of the grain boundaries, the hopping electrons will be stacked and produce polarization at these areas. The electronic conduction is high at low frequencies due to the polarization. However, the electron exchange between Fe^3+^ and Fe^2+^ ions cannot follow the fast alternating field, which decreases the probability of electrons reaching the grain boundaries and the interfacial polarization [19,20]. In Figure 6a,b, the dielectric constant (*ε′*) and dielectric loss (*ε″*) of 3D lamellar graphene/BaFe_12_O_19_ composites enhance with the graphene mass fraction increasing. It is easy to understand that the interlinked graphene sheets can form conducting network to increase the conductivity. With the mass fraction of graphene increasing, the conductivity of the composite improves. Besides, the lamellar graphene/BaFe_12_O_19_/paraffin wax structure has a better dielectric polarization and relaxation effects. When the conductive phase is distributed in the insulating matrix to form composite materials, the free charge gathering will exist in the insulation/conductor interface due to the difference in the two phase conductive performance. The interlinked graphene conductive networks embedded with BaFe_12_O_19_ particles improved the dielectric polarization properties. In Figure 6c,d, it is worth noting that a minimum value as well as a maximum value are observed for *μ′* and *μ″* in some lines, respectively, those obvious peaks for *μ′* and *μ″* can be ascribed to a resonance phenomenon, which is also shown in Figure 7b. From the values of dielectric loss and magnetic loss in Figure 7, it can be concluded that the magnetic loss values of the composites are far lower than their dielectric loss values. Therefore, the main loss mechanism for this composite is dielectric loss rather than magnetic loss.

(1) $$R(dB)=20\log_{10}\left|\frac{Z_{in}-1}{Z_{in}+1}\right|$$

(2) $$Z_{in}={\left(\frac{\mu_{r}}{\varepsilon_{r}}\right)}^{\frac{1}{2}}\tanh\left[j\left(\frac{2\pi fd}{c}\right){\left(\mu_{r}\varepsilon_{r}\right)}^{\frac{1}{2}}\right]$$

where *Z_in_* is the normalized input impedance at free space and material interface, *ε_r_* = *ε′* *− jε"* is the complex permittivity, *μ_r_* = *μʹ − jμ"* is the complex permeability of absorbers, *f* is the frequency of the microwave in free space, *d* is the thickness of the absorber, and *c* is the velocity of light in free space. The impedance matching condition is given by *Z_in_* = 1 to represent the perfect absorption properties. The surface reflectivity of an absorber was presented as a function of six parameters, *ε′*, *ε″, μ′, μ″*, *f* and *d* [21,22]. If the six parameters are known, the absorbing properties of the material can be calculated. The values of reflectivity were calculated by using Equations (1) and (2) with the measured values of *ε′*, *ε″, μ′, μ″, f* and *d* (see Figure 6). In the frequency range 2–18 GHz, 3D lamellar graphene/BaFe_12_O_19_ composites with 30 wt % graphene presents the reflection loss peak at −27.23 dB as well as the frequency bandwidth at 2.28 GHz (<−10 dB). For contrast, under the same matching thickness (*d*_m_ = 2.0 mm), the absorbing peaks of 20 wt % graphene sample presents a slight decrease in absorbing peak and absorbing bandwidth, which is −22.83 dB and 2.04 GHz, respectively. However, when the mass fraction of graphene is up to 40 wt %, the reflection loss and the frequency bandwidth are only 11.18 dB and 1.61 GHz, respectively. Here, a crucial weight value at 30 wt % appears. Based on the former analysis, the dielectric loss tangent of the composite with 40 wt % graphene is larger than that of 20 wt % and 30 wt %. The possible reason for the poor electromagnetic wave absorbing properties of 40 wt % might be the poor matching degree of dielectric loss and magnetic loss, the electromagnetic wave absorbing property of pure EG without BaFe_12_O_19_ was also tested. It can be seen from that the reflection loss peak is only about −5 dB. The high dielectric loss and the poor reflection loss further prove the assumption. Overall, the electromagnetic wave absorbing properties improved with the increasing of graphene mass fraction before 30 wt % and recede after that. The composite with 30 wt % graphene presents the best performance. Figure 8b shows a three-dimensional presentation of the calculated theoretical reflection loss of lamellar graphene/BaFe_12_O_19_ composites at various thicknesses in the frequency range of 2–18 GHz with 30 wt % graphene. This indicates that the microwave absorbing ability of the lamellar graphene/BaFe_12_O_19_ composite at different frequencies can be adjusted by controlling the thickness of the absorbents [23]. With the thickness increasing, the reflection loss peak shift to the lower frequency region. It may be due to the different travel path and the different pass time of incident wave in the different thickness of absorber and the synergistic effect of microwave absorption. Figure 9 shows the electromagnetic wave absorbing mechanism schematics of 3D lamellar graphene/BaFe_12_O_19_ composite. The good electromagnetic wave absorbing properties of 3D lamellar graphene/BaFe_12_O_19_ composite may be explained by the following several reasons. First, the layered structures with high density graphene sheets formed conductive networks leading to more physical contacts between conductive graphene sheets, therefore it increases the resonance circuit density, which is consistent with the good electrical conductivity and large dielectric loss tangent. The graphene possess excellent electrical conductivity. The conducting networks would interact and attenuate the electromagnetic radiation in the absorbers effectively. Second, the polarization relaxation of defects or π-electron and interfacial polarization between graphene and BaFe_12_O_19_ exist. The 3D lamellar graphene structures can consume the incident waves through multiple reflection and scattering inside the layered structures, prolonging the propagation path of electromagnetic waves in the absorbers. The multiple reflections of electromagnetic wave lead to the larger losses of electromagnetic energy. Furthermore, the intertwined layer structures spontaneously and intensely response to the broadband incident wave, presenting as tremendous resistance–inductance–capacitance coupled circuits. The interaction of electromagnetic waves with dielectric materials intensified the molecular motions such as ionic conduction, dipolar polarization relaxation, etc., leading to energy dissipation in the form of heat through molecular friction and dielectric loss and the highly conductive networks would also consume the electromagnetic wave as resistance heat.

## 4. Conclusions

The 3D lamellar graphene/BaFe_12_O_19_ composites were prepared by self-propagating combustion process owing to the synergistic effect of the tremendous heat induced gasification as well as huge volume expansion. The experimental results indicate that 3D lamellar graphene/BaFe_12_O_19_ composites with 30 wt % graphene presents the reflection loss peak at −27.23 dB as well as the frequency bandwidth at 2.28 GHz (≤10 dB) in the frequency range 2–18 GHz. The 3D lamellar graphene nanostructures could consume the incident waves through multiple reflection and scattering inside the layer structures, prolonging the propagation path of electromagnetic waves in the absorbers. The multiple reflections of electromagnetic wave lead to the larger losses of electromagnetic energy.
